# PTSD and Depression Symptoms Increase Women’s Risk for Experiencing Future Intimate Partner Violence

**DOI:** 10.3390/ijerph191912217

**Published:** 2022-09-26

**Authors:** Katherine M. Iverson, Fernanda S. Rossi, Yael I. Nillni, Annie B. Fox, Tara E. Galovski

**Affiliations:** 1 Women’s Health Sciences Division of the National Center for PTSD, VA Boston Healthcare System, Boston, MA 02130, USA; 2 Department of Psychiatry, Boston University School of Medicine, Boston, MA 02118, USA; 3 Center for Innovation to Implementation (Ci2i), VA Palo Alto Health Care System, Menlo Park, CA 94025, USA; 4 Center for Primary Care and Outcomes Research (PCOR), Stanford University School of Medicine, Stanford, CA 94305, USA; 5 School of Healthcare Leadership, Massachusetts General Hospital Institute of Health Professions, Boston, MA 02129, USA

**Keywords:** depression, posttraumatic stress disorder, alcohol use, veterans

## Abstract

Psychological distress may impact women’s risk for future intimate partner violence (IPV). Yet, limited research has utilized longitudinal research designs and there is a scarcity of research looking at the three most commonly implicated mental health factors—posttraumatic stress disorder (PTSD), depression, and alcohol use—within the same study. Research is especially scarce for women veterans, who experience substantial risk for these mental health concerns and experiencing IPV. This study examined the role of PTSD symptoms, depression symptoms, and alcohol use in increasing risk for experiencing future IPV while simultaneously accounting for the impact of recent IPV experience on subsequent mental health. This study included a sample of 1921 women veterans (M_age_ = 36.5), who were asked to complete three mail surveys over the course of 8 months as part of a larger longitudinal survey study of US veterans’ health and well-being. The survey assessed experiences of IPV, PTSD symptoms (PCL-5), depression symptoms (PHQ-9), and alcohol use (AUDIT-C) at each of the three time points. Results from separate path analysis models provided support for the role of PTSD symptoms and depression symptoms (but not alcohol use) in increasing risk for IPV experience over time. However, the path analysis models provided little support, with the exception of PTSD, for the impact of IPV experience on subsequent mental health symptoms. Findings point to the importance of better understanding the mechanisms by which PTSD and depression symptoms can increase risk for IPV to inform theory and prevention and treatment efforts. Detection and treatment of PTSD and depression symptoms among women may help reduce risk for future violence in intimate relationships.

## 1. Introduction

Intimate partner violence (IPV), including physical, psychological, and sexual violence, enacted by a former or current intimate partner, remains one of the most serious and complex population health problems across the globe [1]. This form of violence disproportionally impacts women, as one-third of women worldwide have experienced IPV during their lifetime [1,2]. Similarly, in the United States (US), national surveys indicate that at least one in three women in the US experience IPV during their lifetime [3,4]. It is notable that these prevalence estimates do not include psychological abuse from an intimate partner, which is particularly prevalent and damaging to women’s health [5,6]. Experiences of IPV have far-reaching physical, social, and mental health impacts, including but not limited to physical injuries, somatic symptoms, financial stress, posttraumatic stress disorder (PTSD), depression, substance use disorders, and suicidality [3,7,8,9,10,11].

In addition to these more commonly considered health impacts, individuals who experience IPV are at high risk for experiencing future IPV from the same and/or new partners [12,13]. IPV is often chronic and recurring; over a 6-month period, 22–46% of help-seeking interpersonal trauma (including IPV) survivors report experiencing additional experiences of IPV [14,15]. Unfortunately, whereas IPV is always the responsibility of the individuals who perpetrate the violence, interventions for individuals who use IPV have shown limited effects on long-term reductions in IPV [16,17] and those that are effective [18] are not yet widely disseminated. Therefore, understanding factors that increase women’s risk for experiencing IPV can inform strategies to promote the safety of individuals at risk for future IPV. Although explicating the issue of IPV risk factors has, at times, been criticized as “victim-blaming” [19], empirical work suggests the potential utility in such research in identifying possible prevention and secondary intervention targets to reduce women’s risk for future IPV [8,13,20].

Mental health symptoms, whether from IPV experiences or other stressful experiences, may play a role in increasing risk for IPV experience and maintaining the cycle of abuse [14,21]. However, there is a lack of research examining the temporal associations between specific types of mental health difficulties in contributing to risk for IPV experience using longitudinal designs that involve multiple assessment periods in which both IPV and symptoms are assessed at each time period. The present longitudinal study investigated three mental-health-focused models (PTSD, depression, and alcohol use), examining the temporal relationships between mental health symptoms and experience of IPV among a large community sample of women veterans.

### 1.1. Women Veterans

In the US, the term women veterans refers to women who have served in the military. Women are the fastest-growing group of both active-duty military personnel and veterans and are a population at high risk for IPV [22,23]. Dichter et al. [24] found that women veterans are 1.6-times more likely than non-veteran women to experience IPV during their lifetime. National surveys of women veterans have found that 55% experience IPV at some point during their lifetime [25], while nearly one in four have experienced physical, sexual, and/or psychological IPV within the past year [22,23]. In addition to high IPV prevalence, veterans are an important subgroup for studying IPV risk because they experience high rates of trauma experiences due to military service, namely combat experience and unwanted sexual experiences during military service [26,27], and are at heightened risk for mental health problems [28,29]. Such factors may contribute to their high risk for experiencing recent IPV [30,31].

### 1.2. PTSD Symptoms, Depression Symptoms, and Alcohol Use as Factors Increasing Risk for IPV

The linkages between prior trauma and heightened risk for IPV experience may be due, in part, to mental health symptoms and/or at-risk alcohol use related to these prior experiences. Although the preponderance of research to date has focused on sexual violence as an outcome as opposed to IPV per se, several studies have highlighted the role of mental health symptoms (particularly PTSD, depression, and at-risk alcohol use) as risk factors for subsequent interpersonal violence (e.g., [21,31,32,33,34]). In a systematic review of prospective studies examining mental health symptoms as potential risk factors for future relationship violence among IPV survivors, Kuijpers, Van der Knapp, and Winkel (2011) found some modest support for an effect of PTSD symptoms and alcohol abuse in increasing risk for IPV revictimization, but this research did not find a predictive role of depression symptoms [13]. For example, a longitudinal study of a nontreatment-seeking sample of inner-city women found that PTSD symptom severity, but not depression symptom severity, predicted future IPV above and beyond the effects of previous IPV [35].

More recently, a few longitudinal studies have found support for either PTSD or depression symptoms in increasing risk for future IPV. Among a sample of women seeking help for IPV, PTSD symptoms were found to predict IPV at a 6-month follow-up when controlling for baseline IPV [15]. Additionally, a study of 101 women veterans who experienced past-year IPV found that PTSD symptom severity was positively associated with IPV experience at a 6-month follow-up [36]. Both of these studies were limited to only two time points and neither study examined the association between depression symptoms and future IPV. However, a recent study of women who participated in an intervention for mothers who experienced IPV found depression, but not PTSD, symptoms to increase risk for subsequent IPV [37]. Differences in the conceptualization of IPV may, at least partially, account for these discrepant findings with respect to PTSD. Specifically, Stein et al. [37] examined the total number of abusive partners women reported over 8 years, whereas the Iverson, Litwack et al. [15] and Dardis et al. [36] studies examined behaviorally-specific IPV experiences reported by women at a 6-month follow-up assessment. Additionally, two of these three studies were conducted with women seeking help for IPV [15,37] and may represent specific help-seeking samples. Furthermore, alcohol use was not examined in these studies.

It is possible that women’s use of alcohol may elevate risk for IPV [38]. Although studies have identified alcohol use as a predictor of sexual violence by non-intimate perpetrators [39,40], considerably less research has examined alcohol use as a risk factor for experiencing violence from an intimate partner. One study of women veterans found that higher levels of alcohol use predicted IPV 12 months later, after accounting for baseline IPV experiences and unwanted sexual experiences during military service [41]. However, a prior study with a representative sample of community women aged 18–30 found that alcohol use increased risk for sexual violence by non-intimate perpetrators, but not by an intimate partner [42]. Similarly, a recent study of college women found that alcohol use levels increased risk for sexual assault from non-intimate partners, but did not increase risk for IPV experiences 6 months later [34]. Another study found that higher levels of alcohol use, PTSD, and depression each independently predicted IPV in a large community sample [43], but this was a cross-sectional study, making it impossible to determine if mental health symptoms preceded IPV.

From a theoretical perspective, it is possible that PTSD symptoms, depression symptoms, and alcohol use increase IPV risk through diminished threat detection and use of safety behaviors for women. These mental health symptoms may reduce women’s ability to determine potential threats or risks for violence [14,44,45]. In addition to undermining threat detection, these factors may hinder women’s ability to respond effectively to danger cues from partners and also complicate decision-making and actions necessary to leave abusive relationships. Specifically, it is theorized that symptoms of PTSD, depression, and alcohol use may interfere with help-seeking behaviors and the effective use of available resources that are necessary to curtail future relationship violence [46]. As these factors are important for understanding the risk for IPV, continued research in these domains is needed.

### 1.3. Current Study

To address the aforementioned gaps in the literature, this study conducted secondary analyses from a larger longitudinal survey of veterans’ health and functioning [47] to longitudinally examine whether PTSD symptoms, depression symptoms, and/or alcohol use contributed to experiencing future IPV in a large, diverse, community-based sample of women veterans. This dataset offers a unique opportunity to examine the variables of interest because all were measured in this study and, as noted previously, women veterans represent a critical subgroup of the population that are at high risk for both IPV experiences and mental health symptoms. Yet, there has been minimal attention to the role of mental health symptoms on IPV risk in women veterans and no study has examined together PTSD symptoms, depression symptoms, and alcohol use as potential predictors of IPV in a longitudinal design. Therefore, the current study aims to investigate the influence of each of these mental health factors on future experiences of IPV in a longitudinal cohort of women veterans who completed surveys at baseline (Time 1; T1), a 4-month assessment (Time 2; T2), and an 8-month assessment (Time 3; T3). Based on empirical and theoretical work to date, we hypothesized that PTSD symptoms, depression symptoms, and alcohol use levels would increase risk for IPV experience over time. We evaluated each of these hypotheses in separate models to determine which mental health factors would increase the risk for experiencing IPV over time, while simultaneously accounting for the impact of recent IPV on subsequent mental health and IPV experience.

## 2. Materials and Methods

### 2.1. Participants

This study used data from the first three data collection waves of the Longitudinal Investigation of Gender, Health, and Trauma (LIGHT) study, a national longitudinal mail-based survey of veterans [47]. A primary objective of LIGHT was to examine how community violence experiences impact veterans’ mental health symptoms and how these associations may vary by gender. Thus, LIGHT oversampled for veterans living in high-crime communities as well as women, making this an ideal sample to examine prospective associations between IPV experiences and mental health symptoms among women veterans. Additional sampling details are described in Nillni et al. [47]. In brief, a random sample of veterans between the ages of 18 and 50 was selected from the VA/DoD Identity Repository, a VA-managed database that includes all separated service members. After accounting for non-deliverable addresses (*n* = 8954, 32%), 19,046 veterans were invited to participate (63.8% high crime, 36.2% not high crime) and 3669 veterans enrolled in the study (19% response rate) at Time 1 (T1), of which 1921 (52.4%) identified as women. Given the focus of the present study on women veterans, we examined only those data from women. Women were followed over time with 1304 women participating in Time 2 (T2) (68% response rate) and 1058 women participating in Time 3 (55% response rate).

There were 942 women who participated in all three time periods, 362 women who participated in T1 and T2 only, and 116 women who participated in T1 and T3 only. Women who participated in all three time periods compared to those who participated in only one or two time periods did not differ on variables of interest, including IPV experience, alcohol use, unwanted sexual experiences during military service, and combat experience at baseline. However, women who participated in all three time periods reported fewer PTSD and depression symptoms at baseline compared to those who participated in only T1 and/or T2 (PTSD: M = 25.71, SD = 0.88 and M = 30.72, SD = 0.86, respectively, t(1333) = −4.06, *p* < 0.001) (Depression: M = 6.95, SD = 0.22 and M = 8.62, SD = 0.25, respectively, t(1774) = −5.03, *p* < 0.001).

### 2.2. Procedures

Participants were divided into two cohorts and three mail surveys were administered between August 2018 and August 2019 for cohort A and between February 2019 and February 2020 for cohort B, with approximately four-month intervals in between surveys. Surveys were sent using a modified Dillman [48] mailing strategy with the following procedures at each time point: (a) cover letter, informed consent fact sheet, survey, an opt-out post card, and USD 5 cash pre-incentive; (b) one and half weeks later a thank you/reminder postcard; and (c) 1.5 weeks later an additional reminder letter and survey to non-responders. Veterans who completed the survey received an additional USD 20. The VA Boston Healthcare System Institutional Review Board approved all procedures.

### 2.3. Measures

**Demographic and Military Characteristics**. Information regarding age, race/ethnicity, educational level, relationship status, household income, work status, living situation, and military service information (e.g., branch) were self-reported at T1. Participants also reported on stressful military experiences at T1. Sexual assault during military service was assessed using one item asking about their experience of sexual assault from a non-intimate partner during military service. A positive endorsement of this item was considered positive for unwanted sexual experiences during military service. Combat experience was assessed at T1 using nine items from the Combat Experience Scale and the Aftermath of Battle subscales of the Deployment Risk and Resilience Inventory-2 (DRRI-2; [49]). Items measure experience to combat-related circumstances (e.g., *exposed to hostile incoming fire*) and the aftermath of combat (e.g., *saw civilians after they had been severely wounded or disfigured*). Endorsement of any items was considered positive for combat exposure.

**IPV experience**. IPV was assessed at T1, T2, and T3 using items inquiring about sexual violence (i.e., *unwanted sexual experience by a significant other/spouse (pressured or forced to do sexual things you didn’t want to do)?*), physical violence (i.e., *physical assault (pushed, grabbed, shaken, hit, beat up) by a significant other/spouse?*), and psychological violence (i.e., *emotional mistreatment by significant other/spouse (name-calling, criticized, not allowed to see friends/family, humiliated, or denied money)?*). Items are based on the US Center for Disease Control and Prevention’s definition of IPV experience [50] and were adapted from the Lifetime Trauma Interview for Intimate Partner Violence Survivors [51]. At T1, we used participants’ reports of IPV experience in the past three months. At T2 and T3, participants reported on IPV experience in the past four months. At each time period, participants who positively endorsed at least one of the three items was considered positive for IPV experience in that time period.

**Mental health**. *PTSD symptoms.* At each time period, participants were asked to indicate if they experienced trauma based on a list of traumatic events. Participants that reported trauma were asked to complete the 20-item PTSD Checklist-5 (PCL-5; [52,53]), which assessed PTSD symptoms secondary to their worst trauma. Participants rated the extent to which each symptom bothered them in the past month from 0 (not at all) to 4 (extremely) with scores ranging from 0 to 80. A score of 33 or higher indicates probable PTSD. PTSD symptoms were a continuous measure at each time period. Internal consistency reliability in the sample was excellent (T1 alpha = 0.97; T2 alpha = 0.97; T3 alpha = 0.97).

*Depression symptoms.* At each timepoint, participants completed the 9-item Patient Health Questionnaire (PHQ-9; [54]), which assesses the frequency of depression symptoms in the past two weeks from 0 (not at all) to 3 (nearly every day) with scores ranging from 0 to 27; scores of ≥10 indicate probable depression. We used PHQ-9 scores as a continuous measure of depression symptoms at each time period. The PHQ-9 has demonstrated excellent internal reliability, test–retest reliability, and strong criterion and construct validity [54]. Internal consistency reliability in the sample was excellent (T1 alpha = 0.93; T2 alpha = 0.92; T3 alpha = 0.92).

*Alcohol use.* At each time period, participants completed the Alcohol Use Disorders Identification Test—Consumption (AUDIT-C; [55,56]), which consists of three items inquiring about current alcohol use. The AUDIT-C is a widely used validated screen of risky drinking and alcohol misuse. Each item on the AUDIT-C has a 5-point response option, with total scores ranging from 0 to 12 points. For women, scores of 3 or higher indicate hazardous drinking or having an alcohol use disorder. We used AUDIT-C scores as a continuous measure of alcohol use at each time period. Internal consistency reliability in this sample was acceptable to good at each time period (T1 alpha = 0.69; T2 alpha = 0.66; T3 alpha = 0.87).

### 2.4. Data Analytic Plan

**Figure 1:**
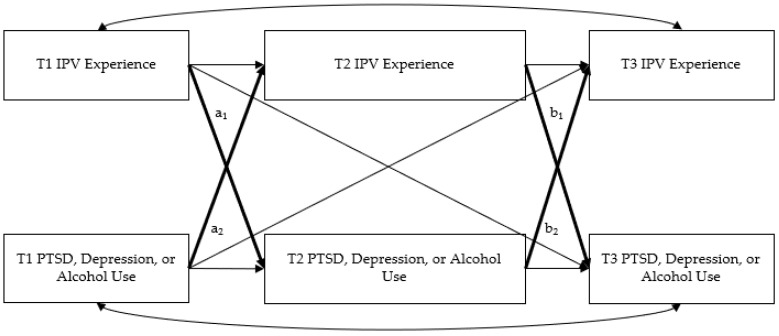
Predicted model examining the bidirectional effects of IPV on mental health outcomes.

First, we computed descriptive statistics and bivariate correlations for the main study variables. Second, we used path analysis to examine the bidirectional effects of IPV experience and mental health symptoms across the three time periods (Figure 1). Models were tested separately for PTSD (PCL-5), depression (PHQ-9), and alcohol use symptoms (AUDIT-C). All models controlled for age, sexual assault during military service, and combat experience given the potential impact of these variables on health outcomes and IPV experience [57,58,59,60]. Models were analyzed using Mplus version 7.3 [61]. Models examined the full sample (1921 women) and missing data were accounted for using full information maximum likelihood estimation. In each model, we allowed all exogenous variables to correlate and included all possible paths. This resulted in just-identified models, making model fit statistics irrelevant. To interpret direct paths, we examined standardized path coefficients and their significance.

## 3. Results

Table 1 displays demographic and military-related characteristics for the T1 sample.

Descriptive statistics and bivariate correlations among variables of interest are displayed in Table 2. At T1, 11.6% (*n* = 214/1843) of the women in the sample reported IPV experience in the past 3 months. At T2 and T3, 14.1% (*n* = 177/1256) and 7.3% (*n* = 74/1012) of women, respectively, reported IPV experience in the past 4 months. Most variables were significantly correlated in the expected directions. However, T2 alcohol use was only significantly and positively correlated with T1 alcohol use (*r* = 0.71). T3 alcohol use was only significantly and positively correlated with T1 alcohol use (*r* = 0.67), T2 alcohol use (*r* = 0.78), and T1 IPV experience (*r* = 0.07).

### Path Models

**Figure 2:**
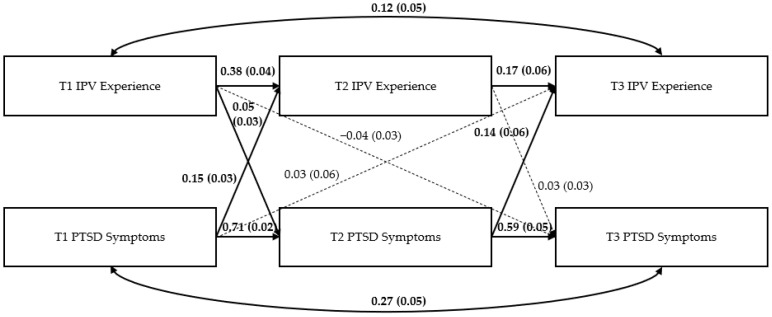
Results of the PTSD model. Note: Numbers represent standardized path coefficients (β) and standard errors (SE) in parentheses. Bolded lines and coefficients are significant; dotted lines are nonsignificant (*p* > 0.05); *n* = 1839 women due to missing data on some variables.

**Figure 3:**
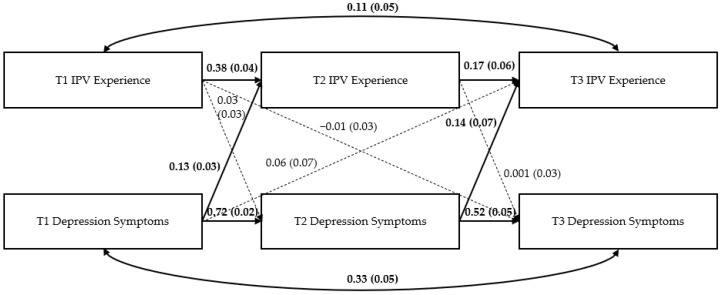
Results of the depression model. Note: Numbers represent standardized path coefficients (β) and standard errors (SE) in parentheses. Bolded lines and coefficients are significant; dotted lines are nonsignificant (*p* > 0.05); *n* = 1842 women due to missing data on some variables.

**Figure 4:**
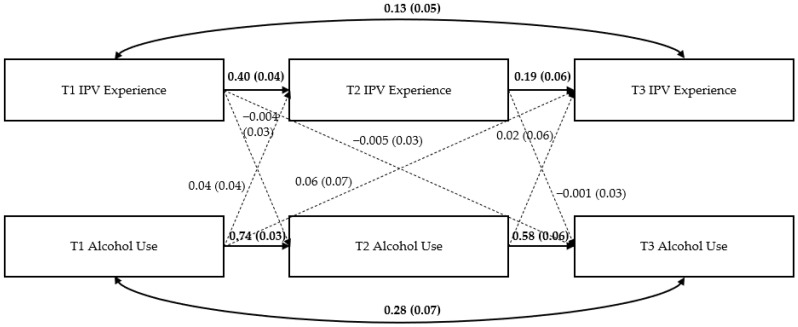
Results of the alcohol use model. Note: Numbers represent standardized path coefficients (β) and standard errors (SE) in parentheses. Bolded lines and coefficients are significant; dotted lines are nonsignificant (*p* > 0.05); *n* = 1841 women due to missing data on some variables.

Figure 2, Figure 3 and Figure 4 show the three path models examining the bidirectional effects of IPV experience and mental health symptoms (i.e., PTSD symptoms, depression symptoms, and alcohol use) across the three time periods. Supplementary Table S1 displays full results for each model.

*PTSD symptom model*. For the PTSD model (Figure 2), there were significant autoregressive effects of T1 IPV experience and T1 PTSD symptoms on their T2 and T3 counterparts (e.g., T1 IPV experience on T2 IPV experience, T2 IPV experience on T3 IPV experience) (all *p* < 0.05). Additionally, T1 PTSD symptoms were significantly and positively associated with T2 IPV experience (β = 0.149, *p* < 0.001) and T2 PTSD symptoms were significantly and positively associated with T3 IPV experience (β = 0.138, *p* = 0.028). There was also a significant and positive association between T1 IPV experience and T2 PTSD symptoms (β = 0.053, *p* = 0.037).

*Depression symptom model*. See Figure 3. Similar to the PTSD model, there were significant autoregressive effects of T1 IPV experience and T1 depression symptoms on their T2 and T3 counterparts (e.g., T1 IPV experience on T2 IPV experience, T2 IPV experience on T3 IPV experience; all *p* < 0.05). Additionally, T1 depression symptoms were significantly and positively associated with T2 IPV experience (β = 0.134, *p* < 0.001) and T2 depression symptoms were significantly and positively associated with T3 IPV experience (β = 0.137, *p* = 0.046).

*Alcohol use model*. See Figure 4. Similar to the PTSD and depression symptom models, results indicated significant autoregressive effects of T1 IPV experience and T1 alcohol use on their T2 and T3 counterparts (e.g., T1 IPV experience on T2 IPV experience, T2 IPV experience on T3 IPV experience) (all *p* < 0.05). However, there were no other significant pathways in this model.

## 4. Discussion

This longitudinal study furthers our understanding of the associations between mental health and IPV risk by examining whether PTSD symptoms, depression symptoms, and alcohol use predict subsequent IPV experience among a large sample of women veterans. Results partially supported our hypotheses. We found that higher PTSD and depression symptoms were both consistently associated with IPV experience over time, whereas alcohol use was not. Although IPV experience was consistently associated with PTSD and depression symptoms in bivariate analyses, IPV was not associated with subsequent depression symptoms or alcohol use in the path analyses. We did find some support for a causal relationship between IPV experience and subsequent PTSD symptoms; however, that relationship only held between T1 and T2, not between T2 and T3. Thus, it appears that prior PTSD and depression symptoms account for a significant amount of the variance in future PTSD and depression symptoms, which may be absorbing any effects of prior IPV experience on mental health in this sample. Moreover, the current pattern of findings is similar to that of another longitudinal study of post-9/11 veterans that found that PTSD symptoms were positively associated with intimate relationship impairment over time, but that relationship impairment was not predictive of increased PTSD symptoms over time [62].

The associations between PTSD symptoms with risk for IPV experience aligns with several studies of IPV survivors that examine associations at only two time points (e.g., [12,15,36]). The present study extends this literature by demonstrating consistent relationships between PTSD and future IPV risk over three time points in a community sample of women veterans, with and without recent IPV experiences. However, it is notable that the current findings are in contrast to those of a longitudinal study of community women that found that PTSD symptoms, but not depression symptoms, predicted future IPV [35]. Similarly, the current findings are inconsistent with findings from two longitudinal studies examining IPV risk among a sample of community women [63] and a sample of women seeking help for IPV [37]. These prior studies found that depression symptoms, but not PTSD symptoms, predicted future IPV [37,63]. In contrast to these prior studies, the current findings suggest that both PTSD symptoms and depression symptoms increase the likelihood of experiencing future IPV. Given the high correlation among depression and PTSD symptoms in our sample, we were unable to include both variables in the same model. As such, it is unclear whether one of these variables is a more important contributor to IPV risk.

It is possible that PTSD symptoms and depression symptoms both contribute to future risk for IPV in different ways. For example, PTSD symptoms may increase risk for IPV due to the occurrence of emotional numbing. This involves a general suppression or analgesia of emotional responsiveness, including anticipatory anxiety associated with danger cues, thereby hindering a woman’s ability to detect risky situations or partners and/or respond to actual risk [12,64]. It is also possible that women with higher levels of PTSD intrusion symptoms, such as heightened emotional distress and physical reactivity in response to trauma reminders, may be more likely to engage in conflict and violence, which may put them at greater risk for experiencing IPV in return [65]. Additionally, alterations in arousal and reactivity, which are characterized by heightened physiological arousal and emotion dysregulation, may lead a woman with PTSD symptoms to be in a constant state of alert. In turn, she may perceive danger and experience high levels of negative emotional arousal (i.e., fear) in various situations, even those that are actually safe. As a result, threat cues lose their prompt value over time and women may come to distrust their physiological and emotional reactions, thereby impeding detection or response to actual threats [15,66].

Depression symptoms may increase risk for IPV experience via a reduced cognitive and affective capacity by which to detect potential abusers and IPV triggers and/or to make decisions to avoid risk. It has also been theorized that depression symptoms may lead women to believe they are not worthy of better treatment, thereby increasing the likelihood of maintaining abusive relationships [14]. Similarly, specific types of depression symptoms, such as worthlessness or hopelessness, may also impede the termination of violent relationships or potentially abusive relationships. Finally, the low motivation and energy levels characteristic of depression may interfere with one’s ability to escape from or seek support for potentially violent relationships [63,67]. Although the exact mechanisms linking depression and PTSD symptoms to IPV experience in this study are unknown, both types of symptoms may cause or exacerbate difficulties in the ability to adequately recognize risk and interfere with safety behaviors in intimate relationships. The current findings reinforce the importance of investigating potential mediators and moderators of these relationships.

Alcohol use was not associated with subsequent IPV experiences in this study, which aligns with findings from two prospective studies conducted with community samples of women and college students [34,42]. Additionally, it is notable that IPV experience was generally not associated with subsequent alcohol use in either the bivariate or path analyses. This is different from the depression symptom findings in the current study. Although IPV experiences were not predictive of subsequent depression symptoms in the path analyses (that simultaneously account for both prior depression symptoms and prior IPV), there were significant and consistent bivariate associations among both recent IPV experiences and depression symptoms concurrently and across subsequent time points. This was not the case for alcohol use. It is possible that IPV experience and alcohol use were not associated due to the study’s limited assessment of IPV. Some evidence suggests that women’s experiences with physical and sexual IPV are more strongly related to higher alcohol consumption than psychological IPV [68,69]. Unfortunately, these types of IPV were not separated in this sample due to their high co-occurrence with psychological violence (i.e., physical and sexual IPV nearly always occur alongside psychological IPV) and the small individual sample sizes for sexual and physical IPV. Future studies should further explore this line of inquiry.

### 4.1. Limitations

There are limitations to this study that can be addressed in future research. First, IPV was a secondary focus of the larger study from which the current data were drawn and, thus, the IPV assessment consisted of three screening items. Although such brief IPV screening items are common in survey research, more comprehensive definitions and assessment of IPV experiences (e.g., intimate partner stalking, control tactics, and reproductive coercion) may have led to more disclosure of IPV experience and more nuanced findings. Future research should include more comprehensive assessments of IPV, such as the Conflict Tactics Scale-2 [70], which includes a wide array of intimate partner physical, psychological, and sexual violence behaviors. Second, in addition to more comprehensive measures of IPV, more rigorous assessments are needed to investigate the impact of important contextual variables associated with IPV, such as women’s perceptions of the impacts of IPV experiences (e.g., presence of fear and control). The Women’s Experiences with Battering Scale [71], which assesses a woman’s perceptions of her vulnerability to physical danger and loss of power and control in an intimate relationship, may provide helpful contextual information and complement other measures of IPV. Third, participants from high-crime areas were oversampled in the larger study and findings may not generalize to the broader population of women veterans or to non-veteran populations. Thus, future studies should attempt to replicate the current findings in representative samples of both women veterans and the general population. Future research also would benefit from the inclusion of additional potential risk factors (e.g., relationship distress/satisfaction and partner characteristics) and mechanisms (e.g., trauma-related beliefs and difficulties with assertiveness in relationships), with an eye toward furthering theory and prevention and intervention development.

### 4.2. Clinical Implications

Identification and effective treatment of mental health symptoms may strengthen efforts to prevent and reduce IPV, but relatively little research to date has specifically focused on this topic [72]. There is evidence that some psychosocial counseling and advocacy-based interventions for women who experience IPV, including samples of veterans, lead to improvements in mental health and possible reductions in IPV experience [73,74,75,76]. In addition, Iverson et al. [14] found that women interpersonal trauma survivors who experienced substantial reductions in PTSD and depression symptoms during cognitive behavioral therapy for PTSD were less likely to report experiencing IPV at a 6-month follow-up compared to women who did not experience similar reductions in these mental health symptoms, adjusting for baseline experiences of IPV. Thus, given the robust associations between these mental health symptoms and subsequent IPV experience observed in this study, evidence-based psychotherapies that target PTSD and depression symptoms may hold promise for reducing women’s risk for experiencing future IPV. Health-care providers may be able to assist women in reducing their risk for IPV through identifying PTSD and depression symptoms and treating resultant mental health symptoms while promoting safety planning and empowerment. This is an important line for future research, particularly among women veterans who experience high risk for both mental health concerns and IPV experience. Brief, motivational-interviewing-based interventions are acceptable and helpful to women veterans and hold promise as a means of increasing mental health treatment initiation and utilization among women veterans at risk for IPV and mental health concerns [75,77,78,79].

These findings have implications for veteran-specific health service settings, a context in which interventions to address IPV are needed [80], including the US Veterans Health Administration (VHA). The VHA is the largest integrated health-care system in the US and treats a rapidly growing population of women veterans [58]. VHA is currently working toward full implementation of IPV screening programs in primary care [81], but the majority of women veteran primary care patients are seen in women’s health mixed-gender and shared-space primary care clinics, where uptake has been low. Yet, VHA patients are routinely screened for both PTSD and depression symptoms in an array of primary care and mental health clinics and elevated symptoms could cue providers to inquire about intimate relationships, including possible IPV behaviors. At-risk women could be referred for services that can address both mental health and relationship concerns. All VA medical centers have an IPV Assistance Program Coordinator (typically a social worker or psychologist) and/or other IPV clinical experts who can meet with patients experiencing or at risk for IPV. These experts can provide information about IPV and options for support services for IPV and mental health in VHA and for IPV services and resources in the community. Findings from this study highlight the continued need for IPV Assistance Program Coordinators and other health-care professionals to provide women with information about mental health resources and services. Such resources and services may not only help reduce mental health symptoms but also protect women from experiencing future IPV.

## 5. Conclusions

Women who suffer from PTSD and depression symptoms experience increased risk for future IPV and such mental health difficulties may make it difficult to prevent or end abusive relationships. Unfortunately, PTSD and depression symptoms are relatively common among women veterans compared to the general population. It is possible that identification and effective treatment of PTSD and depression symptoms among women may help reduce risk for future IPV and interrupt or prevent this cycle of abuse. Although the responsibility for IPV always belongs to those who perpetrate violence, it is important to continue to identify factors that can be intervened upon to reduce women’s risk for IPV experience.

## Figures and Tables

**Table 1:** Sample sociodemographic and military-related characteristics (*n* = 1921).

Characteristics, *M* (*SD*)	*M*	*SD*
Age	36.5	7.6
Characteristics, *n* (%)	*n*	*%*
Race		
White (alone)	1177	62.5
Black (alone)	559	29.7
Asian (alone)	91	4.8
Another/Multiple Races	418	22.2
Hispanic/Latino	228	12
Annual Income		
No income	26	1.4
Less than USD 15k	127	6.9
USD 15k to <USD 25k	189	10.2
USD 25k to <USD 35k	193	10.5
USD 35k to <USD 45k	188	10.2
USD 45k to <USD 55k	189	10.2
USD 55k to <USD 75k	266	14.4
USD 75k to <USD 100k	265	14.4
USD 100k to <USD 150k	242	13.1
USD 150k or more	159	8.6
Relationship Status		
Married	826	43.8
Divorced or separated	496	26.3
Widowed	15	0.8
Never Married	547	29
Education Level		
High school or less	117	6.3
Vocational or technical training	54	2.9
Associates degree or some college	718	38.5
Bachelor’s degree	549	29.4
Graduate or professional degree	430	23
Work Status		
Work for pay (part or full time)	1425	76.7
Homemaker/Caregiver	198	10.7
Retired	69	3.7
Not working	350	18.8
Living Situation		
Own apartment/house	775	41.7
Rent apartment/house/room	912	49
Live with relative/friend	120	6.5
Homeless	11	0.6
Branch of Service		
Army	959	50.6
Marine Corps	102	5.4
Navy	310	16.3
Air Force	491	25.8
Coast Guard	32	1.7
Primary Military Occupation		
Combat Arms	104	5.7
Combat Support	646	35.7
Service Support	1060	58.6
Times Deployed		
0	338	27.9
1	474	39.1
2 or more	399	32.9
Sexual Assault During Military Service	534	28.7
Combat Experience	766	40.3

Note: Missing values possible on each item.

**Table 2:** Descriptive statistics and correlations among variables of interest.

Variable	2	3	4	5	6	7	8	9	10	11	12	*M*	*SD*
1. T1 IPV	0.17 **	0.21 **	0.10 **	0.40 **	0.14 **	0.16 **	0.06	0.21 **	0.12 **	0.16 **	0.07 *	--	--
2. T1 PTSD	—	0.72 **	0.10 **	0.21 **	0.75 **	0.61 **	0.03	0.19 **	0.70 **	0.61 **	−0.01	28.40	22.63
3. T1 Depression		—	0.11 **	0.21 **	0.65 **	0.74 **	0.01	0.21 **	0.64 **	0.71 **	−0.01	7.81	7.03
4. T1 Alcohol use			—	0.09 *	0.04	0.09 **	0.71 **	0.09 *	0.07	0.12 **	0.67 **	3.18	2.20
5. T2 IPV				—	0.23 **	0.25 **	0.05	0.26 **	0.24 **	0.20 **	0.04	--	--
6. T2 PTSD					—	0.77 **	0.02	0.20 **	0.77 **	0.68 **	−0.09 *	22.08	20.24
7. T2 Depression						—	0.04	0.21 **	0.67 **	0.75 **	0.00	6.81	6.50
8. T2 Alcohol use							—	0.04	0.00	0.01	0.78 **	2.30	2.21
9. T3 IPV								—	0.25 **	0.26 **	0.01	--	--
10. T3 PTSD									—	0.79 **	0.00	20.67	20.01
11. T3 Depression										—	0.00	6.52	6.23
12. T3 Alcohol use											—	3.83	3.05

Note: T1 = Time 1; T2 = Time 2; T3 = Time 3; * *p* < 0.05; ** *p* < 0.01; -- = not applicable. The sample size varied due to the differing number of women participating at each time point and missing data. Women who did not participate in the time period of interest or had missing data in that time period were excluded, resulting in sample sizes ranging from 519 to 1728 for correlational analyses.

## Data Availability

The datasets generated during and/or analyzed during the current study are not publicly available due to Human Studies protections placed upon them by the Boston VA Healthcare System Institutional Review Boards. Data are available from the authors upon reasonable request, which would also involve obtaining permission from the VA Boston Healthcare System Institutional Review Board.

## Supplementary Materials

The following supporting information can be downloaded at: https://www.mdpi.com/article/10.3390/ijerph191912217/s1, Table S1: Models predicting the bidirectional associations between IPV experience and PTSD symptoms, depression symptoms, and alcohol use.
