# Supplementary material for: PTSD and Depression Symptoms Increase Women’s Risk for Experiencing Future Intimate Partner Violence
Source: Int J Environ Res Public Health. 2022 Sep 26;19(19):12217. doi: 10.3390/ijerph191912217 (PMC9566456; doi:10.3390/ijerph191912217)
Supplement: Supplementary file 1 [file ijerph-19-12217-s001.zip › ijerph-1906996-supplementary.pdf]

Supplementary Table S1. Models predicting the bidirectional associations between IPV experience and PTSD symptoms, depression symptoms, or alcohol use.

Path	β	SE	*p*-Value
**PTSD Model**			
DV: T2 IPV			
T1 IPV	**0.384**	**0.040**	**<0.001**
T1 PTSD symptoms	**0.149**	**0.034**	**<0.001**
Age	−0.049	0.025	0.053
Combat experience	0.033	0.027	0.219
Sexual assault during military service	0.040	0.032	0.205
DV: T3 IPV			
T1 IPV	**0.118**	**0.051**	**0.021**
T2 IPV	**0.171**	**0.057**	**0.003**
T1 PTSD symptoms	0.033	0.060	0.586
T2 PTSD symptoms	**0.138**	**0.063**	**0.028**
Age	−0.044	0.033	0.177
Combat experience	0.003	0.033	0.924
Sexual assault during military service	0.038	0.036	0.294
DV: T1 IPV			
Age	**−0.067**	**0.024**	**0.005**
Combat experience	0.024	0.024	0.319
Sexual assault during military service	**0.06**	**0.025**	**0.017**
DV: T1 PTSD symptoms			
Age	0.018	0.025	0.463
Combat experience	**0.100**	**0.025**	**<0.001**
MST	**0.286**	**0.024**	**<0.001**
DV: T2 PTSD symptoms			
T1 IPV	**0.053**	**0.025**	**0.037**
T1 PTSD symptoms	**0.714**	**0.022**	**<0.001**
Age	**0.054**	**0.024**	**0.026**

Combat experience	0.041	0.023	0.074
Sexual assault during military service	**0.086**	**0.024**	**<0.001**
DV: T3 PTSD symptoms			
T1 IPV	−0.041	0.025	0.105
T2 IPV	0.025	0.027	0.346
T1 PTSD symptoms	**0.272**	**0.054**	**<0.001**
T2 PTSD symptoms	**0.589**	**0.054**	**<0.001**
Age	0.02	0.028	0.464
Combat experience	−0.011	0.022	0.626
Sexual assault during military service	0.016	0.024	0.512
Covariances			
T1 IPV <--> T1 PTSD symptoms	**0.164**	**0.027**	**<0.001**
T2 IPV <--> T2 PTSD symptoms	0.049	0.035	0.158
T3 IPV <--> T3 PTSD symptoms	0.074	0.050	0.140
**Depression Model**			
DV: T2 IPV			
T1 IPV	**0.381**	**0.041**	**<0.001**
T1 Depression symptoms	**0.134**	**0.031**	**<0.001**
Age	**−0.051**	**0.025**	**0.042**
Combat experience	0.038	0.027	0.155
Sexual assault during military service	0.041	0.032	0.193
DV: T3 IPV			
T1 IPV	**0.108**	**0.051**	**0.034**
T2 IPV	**0.165**	**0.058**	**0.004**
T1 Depression symptoms	0.059	0.065	0.365
T2 Depression symptoms	**0.137**	**0.069**	**0.046**
Age	−0.041	0.033	0.216
Combat experience	0.005	0.032	0.873
Sexual assault during military service	0.027	0.037	0.467
DV: T1 IPV			

Age	**−0.066**	**0.024**	**0.005**
Combat experience	0.024	0.024	0.320
Sexual assault during military service	**0.060**	**0.025**	**0.017**
DV: T1 Depression symptoms			
Age	**0.050**	**0.022**	**0.025**
Combat experience	**0.088**	**0.023**	**<0.001**
Sexual assault during military service	**0.306**	**0.023**	**<0.001**
DV: T2 Depression symptoms			
T1 IPV	0.031	0.025	0.219
T1 Depression symptoms	**0.715**	**0.021**	**<0.001**
Age	−0.028	0.02	0.174
Combat experience	**0.044**	**0.019**	**0.024**
Sexual assault during military service	**0.098**	**0.023**	**<0.001**
DV: T3 Depression symptoms			
T1 IPV	−0.010	0.027	0.722
T2 IPV	0.001	0.027	0.966
T1 Depression symptoms	**0.330**	**0.047**	**<0.001**
T2 Depression symptoms	**0.518**	**0.047**	**<0.001**
Age	0.017	0.022	0.451
Combat experience	**−0.039**	**0.019**	**0.042**
Sexual assault during military service	0.035	0.024	0.139
Covariances			
T1 IPV <--> T1 Depression symptoms	**0.214**	**0.026**	**<0.001**
T2 IPV <--> T2 Depression symptoms	**0.102**	**0.038**	**0.007**
T3 IPV <--> T3 Depression symptoms	**0.092**	**0.046**	**0.044**
**Alcohol Use Model**			
DV: T2 IPV			
T1 IPV	**0.403**	**0.041**	**<0.001**
T1 Alcohol use	0.038	0.036	0.284
Age	−0.039	0.025	0.124

Combat experience	0.043	0.028	0.116
Sexual assault during military service	**0.078**	**0.030**	**0.008**
DV: T3 IPV			
T1 IPV	**0.125**	**0.052**	**0.015**
T2 IPV	**0.194**	**0.059**	**0.001**
T1 Alcohol use	0.061	0.066	0.351
T2 Alcohol use	0.015	0.061	0.805
Age	−0.028	0.034	0.404
Combat experience	0.013	0.033	0.683
Sexual assault during military service	**0.077**	**0.036**	**0.034**
DV: T1 IPV			
Age	**−0.067**	**0.024**	**0.005**
Combat experience	0.024	0.024	0.322
Sexual assault during military service	**0.060**	**0.025**	**0.017**
DV: T1 Alcohol use			
Age	**−0.053**	**0.025**	**0.037**
Combat experience	**0.083**	**0.026**	**0.002**
Sexual assault during military service	**0.092**	**0.029**	**0.001**
DV: T2 Alcohol use			
T1 IPV	−0.004	0.026	0.884
T1 Alcohol use	**0.738**	**0.029**	**<0.001**
Age	0.042	0.022	0.050
Combat experience	−0.013	0.023	0.559
Sexual assault during military service	**−0.072**	**0.025**	**0.003**
DV: T3 Alcohol use			
T1 IPV	−0.005	0.029	0.850
T2 IPV	−0.001	0.030	0.975
T1 Alcohol use	**0.279**	**0.068**	**<0.001**
T2 Alcohol use	**0.576**	**0.059**	**<0.001**
Age	0.023	0.022	0.303

Combat experience	0.025	0.021	0.233
Sexual assault during military service	−0.043	0.023	0.060
Covariances			
T1 IPV <--> T1 Alcohol use	**0.085**	**0.030**	**0.004**
T2 IPV <--> T2 Alcohol use	−0.016	0.040	0.682
T3 IPV <--> T3 Alcohol use	**−0.100**	**0.048**	**0.036**

**Path**	**β**	**SE**	***p*-Value**
**PTSD Model**			
DV: T2 IPV			
T1 IPV	**0.384**	**0.040**	**<0.001**
T1 PTSD symptoms	**0.149**	**0.034**	**<0.001**
Age	-0.049	0.025	0.053
Combat experience	0.033	0.027	0.219
Sexual assault during military service	0.040	0.032	0.205
DV: T3 IPV			
T1 IPV	**0.118**	**0.051**	**0.021**
T2 IPV	**0.171**	**0.057**	**0.003**
T1 PTSD symptoms	0.033	0.060	0.586
T2 PTSD symptoms	**0.138**	**0.063**	**0.028**
Age	-0.044	0.033	0.177
Combat experience	0.003	0.033	0.924
Sexual assault during military service	0.038	0.036	0.294
DV: T1 IPV			
Age	**-0.067**	**0.024**	**0.005**
Combat experience	0.024	0.024	0.319
Sexual assault during military service	**0.06**	**0.025**	**0.017**
DV: T1 PTSD symptoms			
Age	0.018	0.025	0.463
Combat experience	**0.100**	**0.025**	**<0.001**
MST	**0.286**	**0.024**	**<0.001**
DV: T2 PTSD symptoms			
T1 IPV	**0.053**	**0.025**	**0.037**
T1 PTSD symptoms	**0.714**	**0.022**	**<0.001**
Age	**0.054**	**0.024**	**0.026**
Combat experience	0.041	0.023	0.074
Sexual assault during military service	**0.086**	**0.024**	**<0.001**
DV: T3 PTSD symptoms			
T1 IPV	-0.041	0.025	0.105
T2 IPV	0.025	0.027	0.346
T1 PTSD symptoms	**0.272**	**0.054**	**<0.001**
T2 PTSD symptoms	**0.589**	**0.054**	**<0.001**
Age	0.02	0.028	0.464
Combat experience	-0.011	0.022	0.626
Sexual assault during military service	0.016	0.024	0.512

Covariances			
T1 IPV <--> T1 PTSD symptoms	**0.164**	**0.027**	**<0.001**
T2 IPV <--> T2 PTSD symptoms	0.049	0.035	0.158
T3 IPV <--> T3 PTSD symptoms	0.074	0.050	0.140
**Depression Model**			
DV: T2 IPV			
T1 IPV	**0.381**	**0.041**	**<0.001**
T1 Depression symptoms	**0.134**	**0.031**	**<0.001**
Age	**-0.051**	**0.025**	**0.042**
Combat experience	0.038	0.027	0.155
Sexual assault during military service	0.041	0.032	0.193
DV: T3 IPV			
T1 IPV	**0.108**	**0.051**	**0.034**
T2 IPV	**0.165**	**0.058**	**0.004**
T1 Depression symptoms	0.059	0.065	0.365
T2 Depression symptoms	**0.137**	**0.069**	**0.046**
Age	-0.041	0.033	0.216
Combat experience	0.005	0.032	0.873
Sexual assault during military service	0.027	0.037	0.467
DV: T1 IPV			
Age	**-0.066**	**0.024**	**0.005**
Combat experience	0.024	0.024	0.320
Sexual assault during military service	**0.060**	**0.025**	**0.017**
DV: T1 Depression symptoms			
Age	**0.050**	**0.022**	**0.025**
Combat experience	**0.088**	**0.023**	**<0.001**
Sexual assault during military service	**0.306**	**0.023**	**<0.001**
DV: T2 Depression symptoms			
T1 IPV	0.031	0.025	0.219
T1 Depression symptoms	**0.715**	**0.021**	**<0.001**
Age	-0.028	0.02	0.174
Combat experience	**0.044**	**0.019**	**0.024**
Sexual assault during military service	**0.098**	**0.023**	**<0.001**
DV: T3 Depression symptoms			
T1 IPV	-0.010	0.027	0.722
T2 IPV	0.001	0.027	0.966
T1 Depression symptoms	**0.330**	**0.047**	**<0.001**
T2 Depression symptoms	**0.518**	**0.047**	**<0.001**
Age	0.017	0.022	0.451
Combat experience	**-0.039**	**0.019**	**0.042**
Sexual assault during military service	0.035	0.024	0.139
Covariances			
T1 IPV <--> T1 Depression symptoms	**0.214**	**0.026**	**<0.001**
T2 IPV <--> T2 Depression symptoms	**0.102**	**0.038**	**0.007**
T3 IPV <--> T3 Depression symptoms	**0.092**	**0.046**	**0.044**
**Alcohol Use Model**			
DV: T2 IPV			

T1 IPV	**0.403**	**0.041**	**<0.001**
T1 Alcohol use	0.038	0.036	0.284
Age	-0.039	0.025	0.124
Combat experience	0.043	0.028	0.116
Sexual assault during military service	**0.078**	**0.030**	**0.008**
DV: T3 IPV			
T1 IPV	**0.125**	**0.052**	**0.015**
T2 IPV	**0.194**	**0.059**	**0.001**
T1 Alcohol use	0.061	0.066	0.351
T2 Alcohol use	0.015	0.061	0.805
Age	-0.028	0.034	0.404
Combat experience	0.013	0.033	0.683
Sexual assault during military service	**0.077**	**0.036**	**0.034**
DV: T1 IPV			
Age	**-0.067**	**0.024**	**0.005**
Combat experience	0.024	0.024	0.322
Sexual assault during military service	**0.060**	**0.025**	**0.017**
DV: T1 Alcohol use			
Age	**-0.053**	**0.025**	**0.037**
Combat experience	**0.083**	**0.026**	**0.002**
Sexual assault during military service	**0.092**	**0.029**	**0.001**
DV: T2 Alcohol use			
T1 IPV	-0.004	0.026	0.884
T1 Alcohol use	**0.738**	**0.029**	**<0.001**
Age	0.042	0.022	0.050
Combat experience	-0.013	0.023	0.559
Sexual assault during military service	**-0.072**	**0.025**	**0.003**
DV: T3 Alcohol use			
T1 IPV	-0.005	0.029	0.850
T2 IPV	-0.001	0.030	0.975
T1 Alcohol use	**0.279**	**0.068**	**<0.001**
T2 Alcohol use	**0.576**	**0.059**	**<0.001**
Age	0.023	0.022	0.303
Combat experience	0.025	0.021	0.233
Sexual assault during military service	-0.043	0.023	0.060
Covariances			
T1 IPV <--> T1 Alcohol use	**0.085**	**0.030**	**0.004**
T2 IPV <--> T2 Alcohol use	-0.016	0.040	0.682
T3 IPV <--> T3 Alcohol use	**-0.100**	**0.048**	**0.036**

Note. Bolding indicates a significant result. DV = dependent variable; T1 = Time 1; T2 = Time 2; T3 = Time 3; SE = standard error.
